# Is Exercise Protective Against Influenza-Associated Mortality?

**DOI:** 10.1371/journal.pone.0002108

**Published:** 2008-05-07

**Authors:** Chit-Ming Wong, Hak-Kan Lai, Chun-Quan Ou, Sai-Yin Ho, King-Pan Chan, Thuan-Quoc Thach, Lin Yang, Yuen-Kwan Chau, Tai-Hing Lam, Anthony Johnson Hedley, Joseph Sriyal Malik Peiris

**Affiliations:** 1 Department of Community Medicine, School of Public Health, The University of Hong Kong, Hong Kong, China; 2 Department of Microbiology, The University of Hong Kong, Hong Kong, China; U.S. Naval Medical Research Center Detachment/Centers for Disease Control, United States of America

## Abstract

**Background:**

Little is known about the effect of physical exercise on influenza-associated mortality.

**Methods and Findings:**

We collected information about exercise habits and other lifestyles, and socioeconomic and demographic status, the underlying cause of death of 24,656 adults (21% aged 30–64, 79% aged 65 or above) who died in 1998 in Hong Kong, and the weekly proportion of specimens positive for influenza A (H3N1 and H1N1) and B isolations during the same period. We assessed the excess risks (ER) of influenza-associated mortality due to all-natural causes, cardiovascular diseases, or respiratory disease among different levels of exercise: never/seldom (less than once per month), low/moderate (once per month to three times per week), and frequent (four times or more per week) by Poisson regression. We also assessed the differences in ER between exercise groups by case-only logistic regression. For all the mortality outcomes under study in relation to each 10% increase in weekly proportion of specimens positive for influenza A+B, never/seldom exercise (as reference) was associated with 5.8% to 8.5% excess risks (ER) of mortality (P<0.0001), while low/moderate exercise was associated with ER which were 4.2% to 6.4% lower than those of the reference (P<0.001 for all-natural causes; P = 0.001 for cardiovascular; and P = 0.07 for respiratory mortality). Frequent exercise was not different from the reference (change in ER −0.8% to 1.7%, P = 0.30 to 0.73).

**Conclusion:**

When compared with never or seldom exercise, exercising at low to moderate frequency is beneficial with lower influenza-associated mortality.

## Introduction

Physical exercise, as one of a healthy lifestyle, is beneficial in reducing the risk of morbidity and mortality [1], [2]. However, little is known about the mechanism underlying the protective effect of exercise against infectious diseases. Nieman and Nehlsen-Cannarella (1994) have described the relationship between the risk of contracting an upper respiratory tract infection (URTI) and the amount of regular exercise as being a “J-curve relationship” [3], i.e. as the amount of exercise increases, the risk decreases initially but increases after some level. In the few animal studies that have examined the influence of exercise on susceptibility to infectious agents, moderate exercise training prior to infection generally exerts some protection [4], [5], whereas fatiguing or stressful exercise before infection leads to increased mortality [6], [7]. In humans, epidemiological studies assessing exercise and URTI have found that strenuous competitive exercise (e.g. marathon running) leads to an increased susceptibility to URTI [8], [9], whereas moderate training or physical activity can reduce the number of URTI symptoms [10], [11], [12]. But there are no other studies on human subjects. The mechanism of this uncertain health effect of exercise has been explained in terms of a post-exercise change in immunity [13]–[17], however, such change cannot be revealed easily.

The underlying hypothesis of this study is that exercise frequency affects the susceptibility of an adult population (aged 30+) and its majority older subgroup (aged 65+, accounting for 79% of the total) to the activity of influenza viruses in terms of influenza-associated mortality. Based on a database of a large case-control study of lifestyle and mortality (LIMOR study) in 1998 [2] and another database of influenza activity during the same period, we assessed this hypothesis for this population in Hong Kong, a city that represents the region of sub-tropical climates where the influenza activity can be high throughout the whole year. We defined *influenza epidemics* and *influenza intensity* as measures of influenza activities. We performed two kinds of analyses, one on mortality associated with influenza measured by influenza epidemics and the other by influenza intensity. We then compared influenza-associated mortality between exercise groups.

## Results

### Influenza epidemics

**Figure 1:**
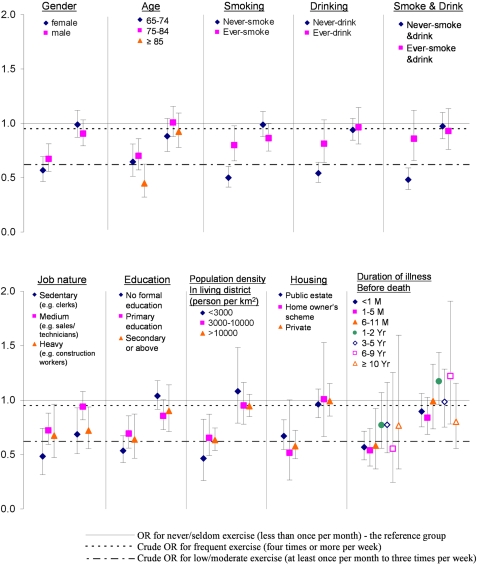
Group specific odds ratios for association between death during influenza epidemic and (i) low/moderate exercise (left) and (ii) frequent exercise (right) relative to never/seldom exercise stratified by social, demographic and life-style factors for age ≥65.

When compared with never/seldom exercisers (Figure 1), the low/moderate exercisers had lower odds of mortality (crude odds ratio 0.62) during influenza epidemics; while frequent exercisers did not show any difference from never/seldom exercisers, with a crude odds ratio close to unity (0.95). There were no substantial differences between the crude odds ratios and the group specific odds ratios stratified by gender, age, education, population density of living area, and housing type. However, obvious differences, where the crude odds ratios were not within the confidence interval ranges of the group specific odds ratios, were shown in groups stratified by smoking, drinking, job nature and duration of illness. These four variables were selected as covariates in calculating the excess risk (ER) by Poisson regression in the second stage.

The difference in ER of mortality (Table 1) during influenza epidemics for low/moderate exercisers relative to never/seldom exercisers in those aged ≥30 ranged from −30% to −37% (P<0.0001 for all-natural and cardiovascular causes; P = 0.003 for respiratory causes) and in ≥65 age group from −38% to −41% (P<0.0001 for all-natural and cardiovascular causes; P = 0.002 for respiratory causes). The ER for frequent exercisers was not significantly (P = 0.28 to 0.96) different from that of the never/seldom exercisers in both age groups.

**Table 1:** Difference in excess risk (ΔER) % (95% C.I.) of mortality during influenza epidemic for low/moderate exercise and frequent exercise relative to never/seldom exercise.

Age group	Causes of death	Low/Moderate	Frequent
≥30	All natural (n = 24,053)	****−29.5 (−37.1, −21.0)	****−2.6 (−10.4, 6.0)
	Cardiovascular (n = 6,587)	****−34.6 (−46.4, −20.1)	**** 0.6 (−13.0, 16.3)
	Respiratory (n = 4,745)	****−37.4 (−53.9, −15.1)	****−1.1 (−18.7, 20.2)
≥65	All natural (n = 18,940)	****−37.9 (−45.9, −28.8)	****−4.9 (−13.1, 4.1)
	Cardiovascular (n = 5,633)	****−39.6 (−51.7, −24.6)	****−0.4 (−14.4, 16.0)
	Respiratory (n = 4,365)	****−40.9 (−57.4, −18.1)	**** 1.1 (−17.2, 23.5)

ΔER% were assessed by multinomial logistic regression, negative values indicate odds reduction vs never/seldom exercise (P-values: ^*^<0.05 ^**^<0.01 ^***^<0.001 ^****^<0.0001); ‘Low/Moderate’ means adults who exercised at least once per month to three times per week, and ‘Frequent’ means adults who exercised four times or more per week.

### Influenza intensity

The ER of mortality associated with each 10% increase in influenza intensity (Table 2) for the never/seldom exercisers ranged from 6% to 9% in the ≥30 age group (P<0.0001) and from 6% to 10% in the ≥65 age group (P<0.0001) in all the mortality outcomes under study. The difference in ER for low/moderate exercisers relative to the never/seldom exercisers in ≥30 age group ranged from −4% to −6% (P<0.001 for all-natural causes; P = 0.001 for cardiovascular causes; P = 0.07 for respiratory causes), and in the ≥65 age group from −6% to −8% (P<0.0001 for all-natural causes; P<0.001 for cardiovascular causes; P = 0.06 for respiratory causes).

**Table 2:** Excess risk (ER) % in the never/seldom exercise group, and difference in excess risk (ΔER)% of mortality associated with each 10% increase in influenza intensity for low/moderate and frequent exercise relative to the never/seldom exercise.

Age group	Causes of death	Never/Seldom (ref.)	Low/Moderate vs ref.	Frequent vs ref.
		[ER% (95% CI)]	[ΔER% (95% CI)]	[ΔER% (95% CI)]
≥30	All natural (n = 24,053)	****5.8 (3.9, 7.6)	****−4.2 (−6.3, −2.0)	**** 0.8 (−1.0, 2.5)
	Cardiovascular (n = 6,587)	****8.5 (5.3, 11.7)	****−6.4 (−10.1, −2.6)	**** 1.7 (−1.5, 4.9)
	Respiratory (n = 4,745)	****6.8 (3.3, 10.3)	****−5.2 (−10.5, 0.4)	****−0.8 (−4.8, 3.4)
≥65	All natural (n = 18,940)	****6.2 (4.1, 8.3)	****−6.1 (−8.6, −3.6)	**** 0.5 (−1.4, 2.5)
	Cardiovascular (n = 5,633)	****9.5 (6.0, 13.0)	****−7.7 (−11.8, −3.5)	**** 1.7 (−1.6, 5.1)
	Respiratory (n = 4,365)	****6.0 (2.2, 9.8)	****−5.5 (−11.0, 0.3)	****−0.7 (−4.8, 3.5)

ER% were assessed by Poisson regression, and ΔER% were assessed by multinomial logistic regression, negative values indicate odds reduction vs never/seldom exercise (P-values: ^*^<0.05 ^**^<0.01 ^***^<0.001 ^****^<0.0001); ‘Never/Seldom’ means adults who never exercised or exercised less than once per month, ‘Low/Moderate’ means adults who exercised at least once per month to three times per week, and ‘Frequent’ means adults who exercised four times or more per week

The difference in ER between frequent exercisers and never/seldom exercisers was not statistically significant (P = 0.30 to 0.73) in both age groups.

## Discussion

Influenza is a major health hazard, contributing to substantial mortality and morbidity with specific patterns in temperate [18], [19], tropical and subtropical climates [20], [21], [22]. Influenza is vaccine preventable [23], however, vaccination coverage is low [24] and the efficacy of vaccination is relatively lower in the older [25] than the younger population [26], [27]. So the adoption of healthy lifestyles which can improve immunity would be effective in the prevention of influenza particularly for older people.

We have shown for the first time with a population based epidemiological perspective that exercising at low to moderate frequency is associated with lower excess risk of influenza-associated mortality (as compared with never or seldom exercise) while exercising frequently does not show any apparent benefit. It appears that a U-shaped dose-response pattern (i.e. as one increases the frequency of exercise, the excess risk of mortality decreases initially until up to some frequency level of exercise then the excess risk increases to that of the never or seldom exercise group) exists for the relationships between levels of exercise frequency and influenza-associated mortality, using influenza intensity as measure for influenza activity, for all-natural, cardiovascular and respiratory causes of mortality. This pattern can be shown when using *influenza epidemics* as measures for influenza activity in comparing the odds of mortality during epidemic periods in three levels of exercise frequency. Such U-shaped dose-response pattern is specific to influenza-associated mortality as shown in this study, but not for effects on mortality per se in another study using the same method to categorize exercise level (Lam et al 2004) [2]. This specificity gives support to inferring causality for the results of our study.

The beneficial effects of exercise on immune function have been explained in terms of viral clearance function [11], [28], [29], antibody titer [30]–[32], and neuroendocrine factors [33]–[35]. However very frequent exercise may not be beneficial. The biological mechanism may be related to exercise-induced inflammation [36] and how much time should be allowed for recovery from the impaired immunity before engaging in another bout of exercise, according to the hypothesis of Pedersen et al (1998) [37]. For those who exercised more than 4 times per week, the duration between two occasions of exercise (less than 2 days) would likely be less than the minimum required period for recovery from the impaired immunity induced by exercise–the ‘*Open Window Theory*’ (Nieman 2000) [38]. Hence during the period of high influenza activity, those who exercised very frequently may have a higher chance of being infected and hence not benefited from the potential protective effects of exercise against influenza, or may even be more vulnerable. Similar explanation could also be applied to the U-shaped patterns of the relationships between air pollution associated mortality and level of exercise, as reported in our earlier study [39]. This explanation remains consistent with the results of Lam et al (2004) [2] when neither air pollution nor influenza, as an external stress on health, is under the assessment, and the U-shaped pattern between exercise frequency and mortality risk was not present [2].

Our results also indicate that during influenza epidemics, the United States recommendation of “*all or most days of the week*” [40] and the United Kingdom recommendation of “*5 or more days of the week*” [41] of doing 30 minutes exercise may be more than is appropriate particularly for older people, and they should not be directly adopted particularly in tropical and sub-tropical regions where influenza activity could be high throughout the whole year. In preparation for emergence of epidemic periods, moderate exercise coupled with increased vaccination coverage may be advisable. Nevertheless we agree with doing more exercises under normal circumstances when influenza intensity is not high.

This study was designed using the influenza activity and lifestyle measuring methods that have been used previously [20], [42] to assess the short term health impact of influenza on people with different lifestyles. Two standard statistical methods have been used: one based on Poisson regression for time-series mortality data, and the other on case-only logistic regression, both of which have recently been applied by us in assessing health effects of air pollution on populations with different lifestyles [39]. In Poisson regression the estimation is robust to whether time independent covariates are included. In this study special attention has been paid to control for time varying confounding factors in that several time dependent covariates including daily averages of temperature, humidity, solar radiation, wind speed and air pollutant concentrations were controlled for so that residual confounding in daily mortality was minimized. The case-only logistic regression is specially designed for assessing interaction between personal and environmental factors on individual case-only data, and the estimation would not be affected by any modeling assumptions for adjustment of the time independent personal and environmental confounding covariates [43].

The study limitations may inevitably include recall errors since the lifestyle data were derived from proxy informants and reflected the habits of the subjects about ten years before death. Nevertheless, we have assessed that the recall error is within an acceptable extent by performing a reliability check with repeated interviews by telephone on a random sample of 235 cases 3 weeks after the initial interview on average. The percentage agreement for exercise as a leisure activity was regarded as satisfactory (73%) [2]. Another criticism could be about possible survivor effects, in that those who were healthier may have been more likely to practice more frequent exercise than those who were in poorer health, and have a generally lower risk profile for mortality from all-natural causes, including respiratory infections. However as most persons aged 30 years or older who died in the study period were included such survivor effects should be minimal. Although air pollution and other environmental factors have been controlled for in the analysis, potential confounding such as vaccination status has not been examined. Besides, the methods used in this study do not rely on a direct linkage between the decedents and the specimens from which the virology data were derived. This would mean that our results could only assess the influenza-associated mortality and not mortality directly due to influenza infection. It is necessary to conduct further studies to confirm our results in other geographic areas and with alternative study design.

## Materials and Methods

### Study Design and Participants

The data in this study were derived from the Lifestyle and Mortality (LIMOR) study, which collected data for adults who died at the age of 30 or above during the period from 1^st^ January to 31^st^ December in 1998. Details of the methods and results on smoking, physical activity and diet have been reported previously [2], [42], [44], [45]. Briefly, the informants, normally one of the more educated relatives of each deceased person, while waiting for the death certificate in one of the four death registries in Hong Kong (as required by the law), were invited and interviewed by a trained interviewer in Cantonese to complete a questionnaire eliciting information on the decedent's socio-demographic characteristics and lifestyle habits including physical exercise about ten years before their deaths. In total 24,656 questionnaires were successfully completed, and 24,053 non-missing cases who died from natural causes were included, accounting for about 80% of all the registered deaths in 1998.

### Mortality Outcomes

For each death, an underlying medical cause was either certified by the registered medical practitioner who attended the deceased during his or her last illness or by the coroner for other deaths. The causes of deaths, coded by the International Classification of Diseases version 9 (ICD-9), were sought from the registries after the interviews and were categorized into all-natural causes (ICD-9 001-799), cardiovascular disease (ICD-9 390-459), and respiratory disease (ICD-9 460-519).

### Influenza Activity

**Figure 2:**
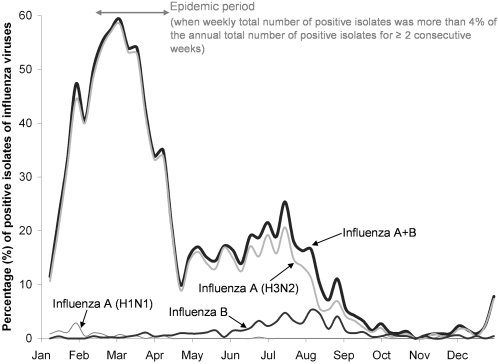
Plots of weekly proportion of total specimens positive for influenza isolation in 1998.

For surveillance of influenza activity, the Public Health Laboratory Service Branch of the Department of Health of Hong Kong Special Administrative Region (SAR) received specimens of respiratory viruses for diagnosis and typing. Culture and subtyping methods were used to detect and identify viruses using WHO reference antisera by haemagglutination inhibition tests. In 1998, there were in total 19,848 specimens for influenza virus isolations collected from all public hospitals (covering about 95% of all hospital beds) and outpatient clinics in the whole territory of Hong Kong SAR. On average, there were 382 specimens collected every week, and the proportion of influenza isolates classified as positive among all collected specimens throughout the whole year was 24.4%, in which the majority was Influenza A subtype H3N2 virus (94%), followed by Influenza B (5%) and Influenza A subtype H1N1 virus (1%). In 1998, the activity of influenza viruses peaked in early March when up to 60% of the weekly specimens were positive influenza isolates (Figure 2). Because of the compact geographical areas and dense population of Hong Kong (total land area: 1,104 km^2^; population: 6.99 million; population density: 6,420 person/km^2^) [46], the data should be representative of influenza virus activity for the whole territory. Throughout the study period, two measures of influenza activity were defined: (i) “influenza epidemic” when weekly numbers of positive isolates of influenza type A (H1N1 or H3N2) and type B, i.e. influenza A+B, were more than 4% of the annual number of positive isolates for two or more consecutive weeks; and (ii) “influenza intensity” as weekly proportion of positive isolates of influenza A+B [20]. For (i), we assigned each death in 1998 as occurring during influenza epidemic or not [20].

### Air Pollution and Meteorological Conditions

Daily average values of air pollution and meteorological data extracted from the Environmental Protection Department (www.epd.gov.hk) and Hong Kong Observatory respectively (http://www.hko.gov.hk/) were used as covariates in statistical models for control of time-varying confounding effects. The descriptive statistics of all daily values for meteorological conditions and air pollution are summarized in Table 3.

**Table 3:** Meteorological conditions and air pollution concentrations in 1998

	mean	min	max
Temperature (°C)	24.0	9.8	31.3
Relative humidity (%)	79.2	38.0	96.0
Solar Radiation (MJ/m^2^)	12.2	0.8	25.3
O_3_ (ìg/m^3^)	32.1	3.9	125.8
PM_10_ (ìg/m^3^)	48.1	15.5	140.5

### Exercise habits

The decedent's exercise habits ten years before their death (around the year in 1988) were asked to avoid any significant chance of the habits being changed by the disease that eventually caused death, so as to minimize reverse causation [2], [47]. The exercise habits were defined according to the number of times per week or month they exercised for 30 minutes or more. The habits were classified into “never/seldom” (n = 16,414): exercised never or less than once per month (E_0_); “low/moderate” (n = 2,852): exercised once per month to three times per week (E_1_); and “frequent” (n = 4,784): exercised at least four times per week (E_2_). *[Note that the original two exercise groups, exercising one to three times per month (n = 1,304) and exercising one to three times per week (n = 1,548), were grouped together due to their insufficient sample sizes for time-series analysis.]* Among all the valid cases, 68% had never/seldom exercised. Those who exercised regularly were more likely to be non-alcohol drinkers, living in private housing, or having a sedentary job (Table 4). They were also more likely to be non-smokers, female, living longer, more educated, or having a better health status before death [39].

**Table 4:** Descriptive statistics of all adults who died at age 30 or above in year 1998

	Never exercise E_0_		Exercise E_1_		Exercise E_2_		Chi-square
	(n = 16,414)		(n = 2,852)		(n = 4,787)		test
	n	%	n	%	n	%	p-value
*Drinking*							
Never drinking	10522	64.5	1953	68.9	3403	71.3	
Ever drinking	5802	35.5	882	31.1	1372	28.7	<0.001
*Population density in living district (no. per km^2^)*							
<3000	1309	8.1	183	6.5	347	7.3	
3000–10000	3634	22.4	676	23.9	1004	21.2	
>10000	11278	69.5	1976	69.6	3382	71.5	0.002
*Housing*							
Public estate	7587	56.8	1228	47.9	2083	50.7	
Home owner's scheme	689	5.2	179	7.0	252	6.1	
Private whole	5074	38.0	1155	45.1	1777	43.2	<0.001
*Job nature*							
Sedentary (e.g. clerks)	1833	14.0	506	22.0	630	17.3	
Medium (e.g. sales/technicians)	8609	65.5	1443	62.7	2420	66.4	
Heavy (e.g. construction workers)	2696	20.5	351	15.3	593	16.3	<0.001

E_0_ = never/seldom; E_1_ = low/moderate; E_2_ = frequent

### Statistical Analysis

#### Influenza epidemic

For the association between influenza epidemic and exercise habit, the number of E_1_ or E_2_ versus E_0_ exercisers were cross classified, according to whether they died in an epidemic or non-epidemic period, into two 2×2 tables respectively. Then odds ratios for E_1_ or E_2_ against E_0_ were computed and they were termed as the “crude odds ratios”, which were then further stratified to “group specific odds ratios” by gender, age group, smoking, drinking, job nature, educational attainment, population density of the decedent's living district, housing type, and the duration of illness before death to assess the association between frequency of exercise and death in influenza epidemics to different personal characteristics and risk factors. For the difference in excess risks (ER) of mortality associated with influenza epidemics between exercise groups (between E_1_ and E_0_, or E_2_ and E_0_ ), multinomial logistic regression under the case-only approach [43], [48] was applied by using the variable that indicated the three exercise groups as the categorical dependent variable and the influenza epidemic indicator as the independent variable (X).
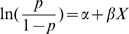
where *p* = probability that one is having a particular exercise habit. The influenza effect which may be modified by an exercise habit is estimated by (exp(*β*)−1)×100%.

#### Influenza intensity

For ER of mortality associated with influenza intensity among the never/seldom exercise group, the weekly proportion of positive isolation of influenza A+B (influenza intensity) as the independent variable and the daily counts of death as the dependent variable were fitted to obtain the core model using generalized additive model [49]. The core model was adjusted for seasonality (defined by a natural spline smoothing function with 2–6 degrees of freedom), temperature, humidity, solar radiation, ambient air concentration including particulate matter with an aerodynamic diameter of 10 micrometers or smaller (PM_10_) and ozone (O_3_). The potential confounding factors also adjusted for were proportion of smokers, drinkers, subjects with illness for one year or more before death, and having a sedentary or heavy job; as well as dummy variables for holiday and day of the week. The core model was regarded as adequately controlled for confounding if the partial autocorrelation function plots for the residuals were less than 0.1 and were free from any systematic patterns. For difference in ER of mortality associated with influenza intensity between exercise groups (between E_1_ and E_0_, or E_2_ and E_0_), multinomial logistic regression was applied by using the categorical exercise group variable as the dependent variable and the influenza intensity as the independent variable. All ER values obtained from the regression models were transformed as a percentage per 10% increase of influenza intensity, i.e. the influenza effect which may be modified by an exercise habit is estimated by (exp(10×*β*)−1)×100%. All analyses were performed by using the R 2.4.0 program.
